# Reassessing the Standard Chemotaxis Framework for Understanding Biased Migration in *Helicobacter pylori*

**DOI:** 10.1146/annurev-chembioeng-100722-114625

**Published:** 2024-07-03

**Authors:** Jyot D. Antani, Aakansha Shaji, Rachit Gupta, Pushkar P. Lele

**Affiliations:** 1 Artie McFerrin Department of Chemical Engineering, Texas A&M University, College Station, Texas, USA; 2 Current affiliation: Department of Ecology and Evolutionary Biology, Center for Phage Biology & Therapy, and Quantitative Biology Institute, Yale University, New Haven, Connecticut, USA; 3 Department of Biomedical Engineering, Texas A&M University, College Station, Texas, USA

**Keywords:** chemotaxis, polarly flagellated bacteria, flagellar motor, switching, phosphorylated CheY, rotational bias

## Abstract

*Helicobacter pylori* infections are a major cause of peptic ulcers and gastric cancers. The development of robust inflammation in response to these flagellated, motile bacteria is correlated with poor prognosis. Chemotaxis plays a crucial role in *H. pylori* colonization, enabling the bacteria to swim toward favorable chemical environments. Unlike the model species of bacterial chemotaxis, *Escherichia coli*, *H. pylori* cells possess polar flagella. They run forward by rotating their flagella counterclockwise, whereas backward runs are achieved by rotating their flagella clockwise. We delve into the implications of certain features of the canonical model of chemotaxis on our understanding of biased migration in polarly flagellated bacteria such as *H. pylori*. In particular, we predict how the translational displacement of *H. pylori* cells during a backward run could give rise to chemotaxis errors within the canonical framework. Also, *H. pylori* lack key chemotaxis enzymes found in *E. coli*, without which sensitive detection of ligands with a wide dynamic range seems unlikely. Despite these problems, *H. pylori* exhibit robust ability to migrate toward urea-rich sources. We emphasize various unresolved questions regarding the biophysical mechanisms of chemotaxis in *H. pylori*, shedding light on potential directions for future research. Understanding the intricacies of biased migration in *H. pylori* could offer valuable insights into how pathogens breach various protective barriers in the human host.

## INTRODUCTION

Motility is a remarkable product of evolution that allows living organisms to quickly inhabit new territories. The ability to move significantly enhances the chances of survival, which is why motility is common across all domains of life (1). It is especially widespread across the bacterial kingdom. Bacteria exhibit numerous forms of motility, including gliding, sliding, twitching, flagellar motility, and swarming (2, 3). By far, the fastest is flagellated motility, enabling a cell to cover distances 10–50 times its own length in a second (4). Flagellated motility greatly enhances the rate at which a bacterial population can spread in a niche, almost as quickly as sugar molecules, surpassing the rate at which nonmotile bacteria spread by several orders of magnitude (5–7). Not surprisingly, a significant fraction of known bacterial species carry flagellar genes (8, 9).

Flagellated bacteria rotate extracellular flagellar filaments with the aid of tiny electric motors known as flagellar motors (10). The rotation of the helical filaments exerts a propulsive force on the cell body, enabling it to move in a persistent direction for a few seconds through highly viscous environments. During the course of evolution, the flagellar motor likely became coupled to a signaling network capable of controlling flagellar rotation (11). Modulation of rotation influenced the random movements of cells, biasing their migration toward favorable chemical environments and further enhancing their chances of survival (12). This phenomenon, known as chemotaxis, has been studied extensively in the model species *Escherichia coli* (13, 14). However, flagellated bacteria exhibit a wide diversity of motility patterns and underlying mechanisms of flagellar modulation. This diversity limits the applicability of the canonical model in explaining directed migration, specifically in polarly flagellated species (6). Here, we delve into some of these limitations with a focus on the opportunistic pathogen *Helicobacter pylori*.

## MODULATION OF FLAGELLAR ROTATION IN THE CANONICAL CHEMOTAXIS NETWORK

**Figure 1:**
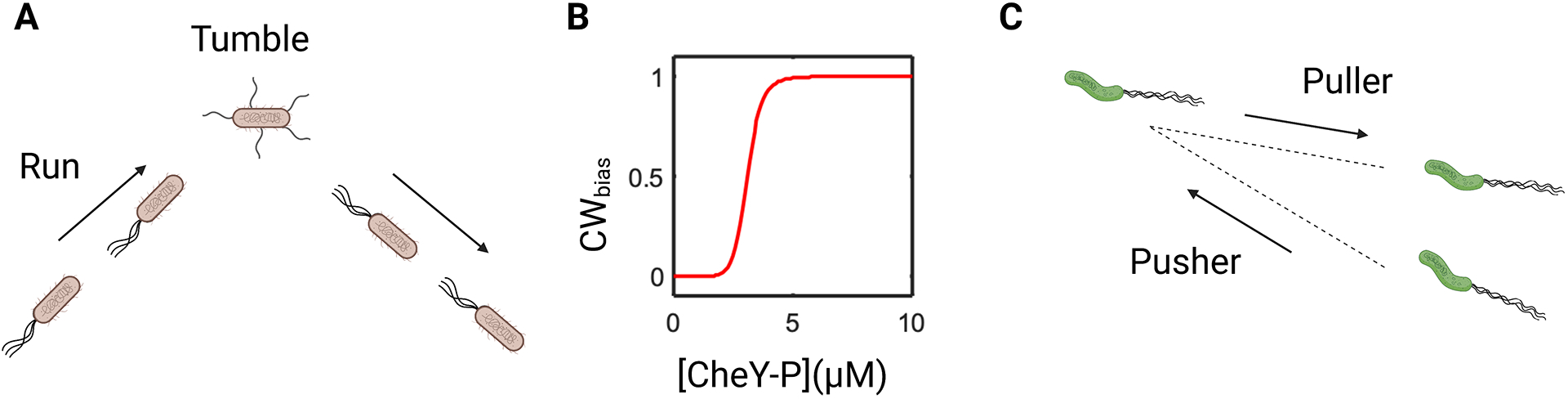
The canonical model of chemotaxis and motility patterns. (*a*) A cell of *Escherichia coli* runs by rotating its flagella counterclockwise (CCW). A switch to clockwise (CW) rotation in one or more flagella causes a tumble. When all the flagella resume CCW rotation, the cell initiates another run. (*b*) The experimentally determined CW bias (probability of CW rotation) versus phosphorylated CheY (CheY-P) levels is a sigmoidal relationship with a steep gradient (Hill coefficient ~10–20). (*c*) In *Helicobacter pylori* cells, the flagella are localized at the pole. CCW rotation of the flagella causes cell to swim in the pusher (forward) mode, and CW rotation causes it to swim in the puller (backward) mode. Figure adapted from images created with BioRender.com.

Individual cells of *E. coli* carry approximately three to four left-handed flagellar filaments that rotate counterclockwise (CCW) by default. CCW rotation causes the filaments to bundle together (Figure 1*a*), propelling the cell in a relatively straight path known as a run. The chemotaxis network induces stochastic switches in the direction of rotation of the flagella between CCW and clockwise (CW). When filaments rotate in the opposite directions, the bundle comes apart, causing the cell to tumble (15, 16). The cell navigates its environment by alternating between runs and tumbles (17).

The chemotaxis-signaling network in *E. coli* is composed of transmembrane methyl-accepting chemotaxis receptors that detect extracellular ligands. The receptors then modulate the activity of a chemotaxis sensor kinase, CheA. In turn, CheA modulates the direction of rotation of the flagellar motor by controlling the phosphorylation levels of a response regulator called CheY. An increase (decrease) in CheA activity increases (decreases) phosphorylated CheY (CheY-P) levels. CheY-P binds to two complexes, called FliM and FliN, at the base of the flagellar motor. CheY-P binding increases the probability of CW rotation, also known as the CW bias (18). The effect of CheA activity on the CW bias has been explained successfully based on a thermal isomerization model (19, 20).

The CW bias is highly sensitive to variations in CheY-P levels in *E. coli*, as characterized by a sigmoidal curve with a Hill coefficient of approximately 10–20 (21–23) (Figure 1*b*). Even a small change in the activity of CheA, and hence, CheY-P levels, can saturate the motor response, inducing CCW-only or CW-only rotation. The cell maintains the basal CheA activity at a value that ensures a baseline value of CW bias ~0.1–0.5. When the cell is exposed to a chemo-effector or a ligand that it prefers, known as a chemo-attractant, or simply an attractant, CheA activity decreases. This decreases the CW bias. Conversely, when the cell is exposed to a ligand that it does not prefer, termed a chemo-repellent or repellent, the CheA activity and the CW bias increase. Thus, the CW bias is a sensitive probe of the CheA activity and the response of the cell to different ligands (24).

To continue swimming up or down a chemical gradient, the cell must continually reset the CW bias back to its basal value. This allows for a continuous response to the constantly fluctuating chemical signal. The resetting or adaptation of CW bias is accomplished mostly through the action of two enzymes, CheR and CheB. CheR is a methyltransferase that adds methyl groups to the glutamate side-chain residues on the receptors, elevating CheA activity. CheB, when phosphorylated (CheB-P), functions as a methylesterase. It demethylates the receptors to decrease CheA activity (25). The addition of methyl groups by CheR is a relatively slow process compared to the removal of methyl groups by CheB-P (26). The two enzymes work in tandem on the receptors to ensure that steady-state CheY-P levels are maintained within the motor’s sensitive range (i.e., 0 < CW bias < 1).

Another mechanism for adapting the CW bias involves the remodeling of motor complexes, FliM and FliN, that bind CheY-P (23, 27). This remodeling process is slower, taking several minutes, and is secondary to the rapid adaptation achieved through receptor modification, which occurs within a few seconds. Although motor remodeling contributes to overall performance (28), it is primarily receptor modification that drives adaptation in CW bias. The precise adaptation in CW bias is crucial for expanding the dynamic detection range and improving the network’s ability to respond to signals that change over time.

## MOTILITY PATTERNS: RUN-AND-TUMBLE VERSUS RUN-AND-BACKWARD RUNS

*E. coli* cells possess flagella that are uniformly distributed on their surfaces. Consequently, local concentrations of CheY-P can vary at the sites of different motors within the same cell. Such concentration variations and the fact that flagellar switching is probabilistic mean that the motors on a cell may not switch simultaneously despite the steep relationship between CW bias and CheY-P (29). Consequently, one or more filaments will often rotate in opposite directions, causing frequent tumbles.

In contrast, polarly flagellated cells localize all their flagella at the poles. Several bacterial species, such as *Caulobacter crescentus*, *Vibrio parahaemolyticus*, and *Pseudomonas aeruginosa*, carry a single polar flagellum, whereas others, such as *Vibrio fischeri*, *Pseudomonas putida*, and *H. pylori*, carry multiple flagella at their poles (30–33). Unlike randomly distributed flagella that lead to a run-and-tumble pattern of motility, polar flagella tend to give rise to a run-and-backward run pattern of motility. The run occurs when the motor rotates in one direction, thrusting the cell forward, with its flagellum lagging behind the body. This is termed the pusher mode. The backward run occurs when the motor rotates in the opposite direction, causing the cell to move back, with the flagellum leading the cell body. This is termed the puller mode (Figure 1*c*). Whether CCW rotation corresponds to pusher or puller mode depends on the handedness of the flagellar filament. For example, CCW flagellar rotation causes *P. aeruginosa* to swim in the pusher mode because the flagella are left-handed, whereas CCW rotation causes *C. crescentus* to swim in the puller mode because their flagella are right-handed (34–36). In some bacterial species, buckling of the universal joint connecting the flagellar filament and the flagellar motor, called the flagellar hook, can cause the motility pattern to deviate from run-reversal to a run-flick-reverse or other forms (37, 38).

We reported recently that *H. pylori* cells appear to switch between puller and pusher modes without any discernible flicks. The close proximity of the flagella at a single pole suggests that the local concentrations of CheY-P at the different motors are relatively similar. This uniformity in concentration likely facilitates synchronized switching, leading to nearly instantaneous changes in movement direction, either forward or backward. The absence of tumbling in these bacteria makes it challenging to extend the general chemotaxis framework to this particular species, which we explore further below.

## POTENTIAL ISSUES WITH EXTENDING THE CANONICAL CHEMOTAXIS FRAMEWORK TO POLAR FLAGELLATES

**Figure 2:**
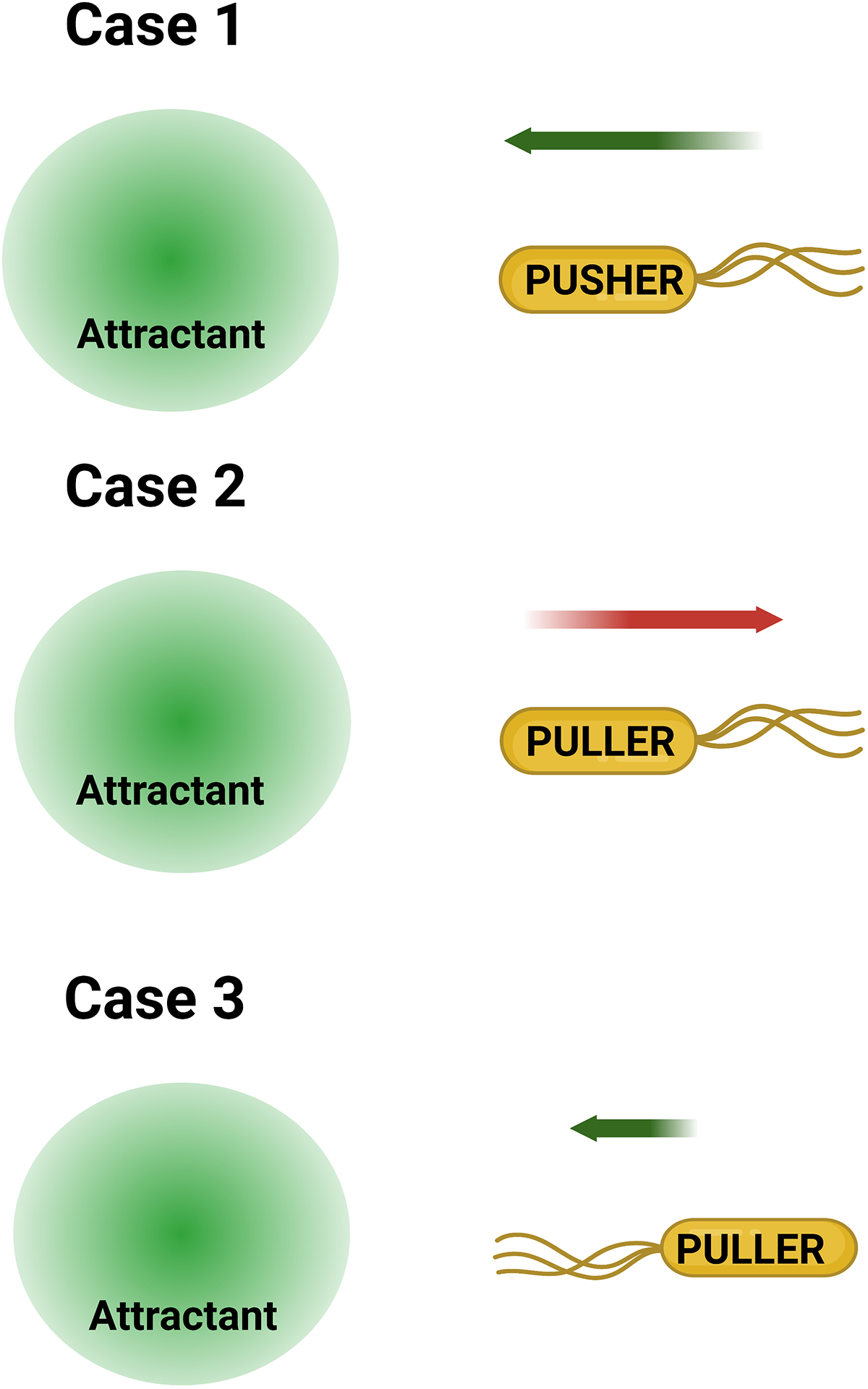
Examination of predicted chemotaxis errors. Case 1: A pusher experiences higher attractant levels as it moves toward the source. The consequent falling kinase activity promotes counterclockwise (CCW) flagellar rotation, sustaining the pusher mode. This enables extension of the cell’s run in the appropriate direction. Case 2: A puller experiences lower attractant levels as it moves away from the source. The increasing kinase activity promotes CW flagellar rotation, sustaining the puller mode. As a result, the cell migrates in the wrong direction away from the attractant source. Case 3: A puller experiences increasing attractant levels as it backs into the source. The falling kinase activity promotes CCW rotation and, hence, the pusher mode. This increases the probability that the cell’s movement in the appropriate direction is terminated prematurely. The red and green arrows indicate movement in the wrong and correct directions, respectively. Their lengths indicate the duration of travel. Figure adapted from images created with BioRender.com.

When an *E. coli* cell encounters an attractant, CheA activity decreases, depressing the probability of CW bias. This results in an increase in the mean run time (τ _*pusher*_), prolonging the cell’s current run along the attractant gradient (Figure 2, case 1). Conversely, when the cell moves down the gradient, the diminishing attractant levels increase CheA activity. Elevated CheA activity increases CW bias, promoting a tumble. Because the cell does not go any further down the gradient owing to the tumble, it has time to adapt its CW bias and initiate a new run. Thus, the tumbles occur with different frequencies—the number of times a cell tumbles per unit time—depending on the direction of the swimmer’s run with respect to the gradient (12).

A comprehensive picture of flagellar modulation under varying chemo-effector levels has been developed from single-motor experiments (24, 39). A recurrent finding is that a step-decrease in the attractant concentration can induce CW-only rotation in single flagellar motors for a few seconds prior to adaptation. A step-increase in the concentration of indole, a powerful repellent, induces a similar effect for much longer durations (40). Ultimately, the flagellar motor tends to rotate in the CW direction for extended periods when a stimulus, such as decreasing attractant concentration, causes CheA activity to increase significantly.

Biased migration in a polar flagellate resembles that in *E. coli* for the scenario in which a cell swims up a chemoattractant gradient in the pusher mode. As attractant concentrations increase, CheA activity diminishes, increasing *τ*_*pusher*_. This extends the cell’s movement up the attractant gradient (Figure 2, case 1). However, the eventual reversal to the puller mode tends to negate the spatial gains made during the pusher mode. This is especially true in the absence of flagellar flicking or kinematic oddities, as the cell may back up at an angle of ~180° relative to its original direction of travel (6).

Significantly, the puller mode could give rise to errors between the chemotaxis signaling feedback and the physical movements of the cell. The cell’s ability to swim in the puller mode, as opposed to tumbling, means that the cell may occasionally back away or back into the source of a chemo-attractant. Consider the case of a polarly flagellated cell that moves in the pusher mode by rotating its motors CCW and in the puller mode by rotating its motors CW. In the hypothetical scenario depicted in Figure 2 (case 2) that may arise due to random chance, the cell’s movement in the puller mode from areas of high to low attractant concentrations causes the receptors to detect a decline in the number of attractant molecules. This decrease in attractant concentration enhances kinase activity, elevating CheY-P levels, as detailed above. If the cell modulates its flagellar functions in a manner similar to that in a cell of *E. coli*, the increased CheY-P binding to the motor will boost *τ*_*puller*_. In this scenario, the cell will continue migrating away from the attractant source, creating an error in the direction of migration.

In another scenario arising by random chance, in which the cell attempts to back into the attractant source in the puller mode, the rise in attractant levels due to cell motion tends to suppress CheA activity. This increases the probability of a switch to the pusher mode, causing the cell to prematurely terminate its motion toward the attractant (Figure 2, case 3). The eventual run in the pusher mode will be terminated just as quickly, owing to increasing CheA activity.

Thus, a polarly flagellated bacterium capable of swimming in the puller mode has an equal likelihood of swimming up or down an attractant gradient. In predicting these errors, we made several implicit assumptions, including rapid swimming speeds, a steep chemical gradient, a significant gain and an instantaneous response of the network to the external stimuli, and slow or absent adaptation in kinase activity. These are reasonable assumptions based on what is already known about motility in polar flagellates, such as *H. pylori*, and the mechanism by which the chemotaxis network in *E. coli* modulates flagellar functions.

## MODULATION OF REVERSAL FREQUENCY CORRECTLY PREDICTS CHEMOTAXIS IN RUN-TUMBLE AND RUN-BACKWARD RUN SPECIES

The directional movement of an *E. coli* cell undergoing runs and tumbles is uncorrelated over long times. The bacterial population’s dispersion can then be described by an effective diffusion coefficient (41), which scales as the square of the cell’s swimming speed and is proportional to *τ*_*pusher*_. It also depends on the angle of reorientation of the cell during a tumble or, in other words, the persistence in its directional motion. On average, a tumble causes the bacterium to reorient itself by ~70° relative to its original direction of travel (5).

In the presence of an attractant field, a chemotactic drift is superimposed that biases the cell’s otherwise random walks. This drift causes an increase in cell density near the source of the attractant over time. The mean velocity at which bacteria drift up the concentration gradient is influenced greatly by the specific mechanism through which the chemotaxis signaling network ultimately modulates flagellar functions. For example, the cell could modulate its tumbling frequency, swimming speed, or turning angle (42). In the case of *E. coli*, the network primarily modulates the CW bias to bias cell migration. The accompanying changes in the tumbling frequency are reflected in the unimodal dependence of the flagellar motor’s switching frequency on CheA activity (20, 21).

Because the cell does not undergo translational displacement during a tumble, it is not necessary to explicitly consider tumble duration when modeling cell migration. In fact, several models have been proposed that successfully reproduce experimental measurements of chemotaxis by assuming that *τ*_*pusher*_ varies based on the chemical gradient (43–47), without explicitly accounting for variations in tumble duration or CW bias modulation.

In the case of polarly flagellated bacteria, however, both *τ*_*pusher*_ and *τ*_*pusher*_ produce cell displacement. To our knowledge, modeling studies on the migration of polarly flagellated bacteria are limited; however, existing works have modeled the dispersion and chemotaxis of the cell population by assuming that the cell modulates reversal frequencies or swimming speeds (6, 31, 48–51). The reversal frequency refers to the number of times the cell alternates between pusher and puller modes in unit time. If the chemotaxis signaling network in these cells indeed increased the reversal frequency, rather than the CW bias, through CheA activity, the two error-prone scenarios depicted in Figure 2 (cases 2 and 3) would be resolved: The cell would revert to the pusher mode before moving too far in the wrong direction.

## THE CHEMOTAXIS NETWORK IN *HELICOBACTER PYLORI* MODULATES CLOCKWISE BIAS

How valid is the assumption that the chemotaxis network in polar flagellates modulates reversal frequency? In *Vibrio* and *Pseudomonas* species, the limited data suggest that the cells mostly modulate their reversal frequencies (50, 52), consistent with the aforementioned optimal strategy for chemotactic navigation in polarly flagellated species. Notable gaps exist in our understanding of flagellar modulation due to the technical difficulties in investigating its mechanisms. Being no more than a few nanometers in diameter, the flagella are challenging to visualize. This makes it difficult to distinguish between puller and pusher modes during chemotaxis. In peritrichous polar flagellates, probing single motor function is particularly challenging because these filaments are closely spaced (6).

In our recent work, we observed that *H. pylori* cells swim at different speeds in the puller and pusher modes. Our dispersion modeling results indicated that this asymmetry in swimming speed enabled *H. pylori* to spread much faster compared to *E. coli* in 3D space. We further exploited this asymmetry in swimming speeds to quantify the CW bias by analyzing the swimming trajectories of individual cells. This approach proved beneficial because visualizing and probing the flagella directly are technically challenging in *H. pylori*. Further, the cells rotated their flagella CCW when swimming in the pusher mode and instantaneously reversed their paths by transitioning into the puller mode via CW flagellar rotation. We found no evidence supporting the occurrence of a run-reverse-flick behavior (6).

From the asymmetric tracks, we calculated that the basal CW bias was ~0.35 in wild-type cells, showing a preference for swimming in the pusher mode. The CW bias and reversal frequency decreased when the cells were treated with a potent attractant, urea. The CW bias was zero in a mutant that lacked the chemotaxis response regulator, CheY. This strongly suggested that the default direction of flagellar rotation is CCW and that CheY-P interactions with the flagellar motor increase the CW bias. Thus, *H. pylori* is an intriguing exception among the few polar flagellates that have been tested, in that it appears to adopt a strategy that mirrors *E. coli*’s strategy of modulating flagellar functions (6).

**Figure 3:**
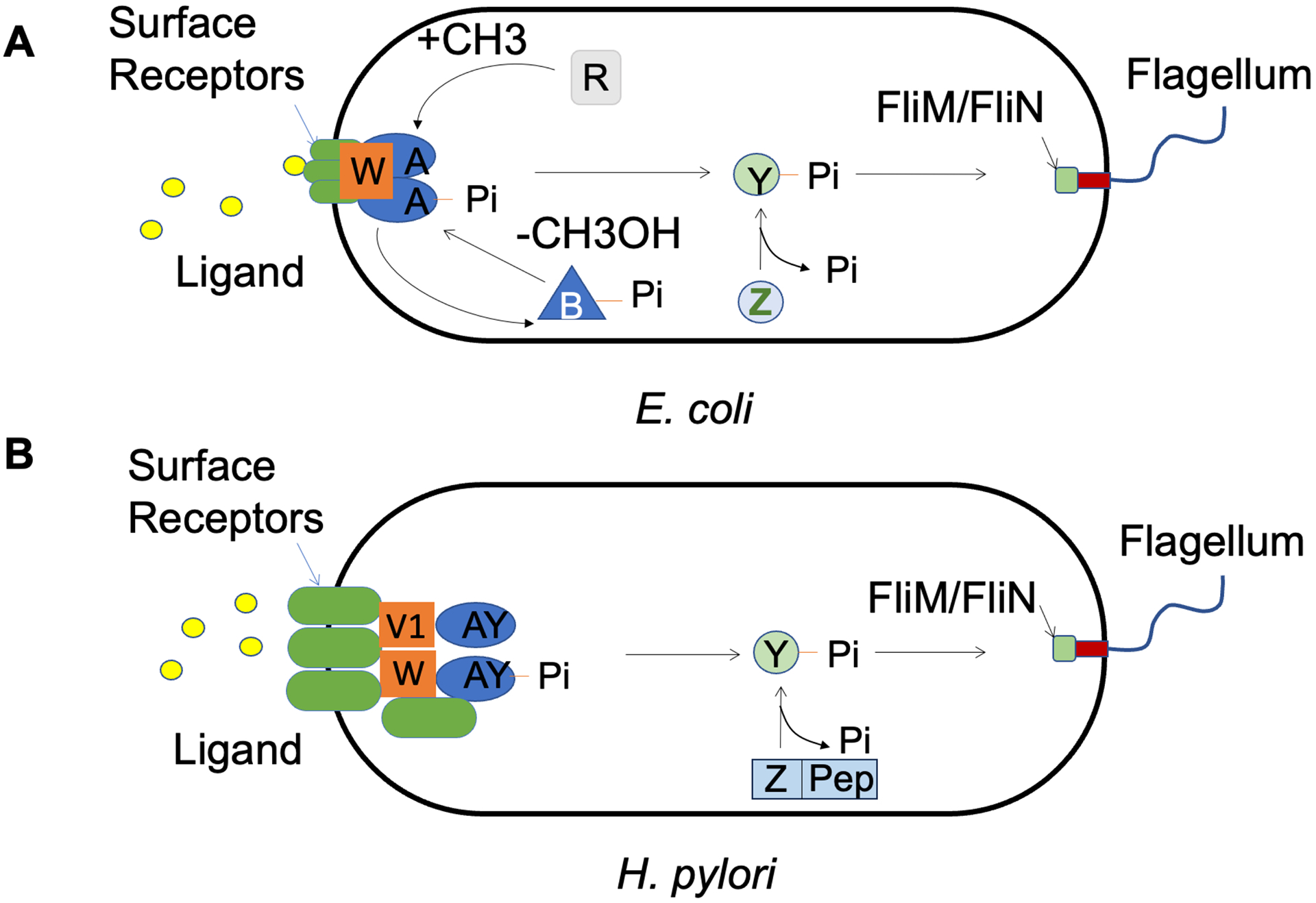
Comparison of the chemotaxis networks in (*a*) *Escherichia coli* and (*b*) *Helicobacter pylori*. A cascade of reactions controlled by the membrane-embedded chemoreceptors modulates flagellar reversals in both species. Chemotaxis (Che) proteins are denoted by the letters at the end of their names. The freely diffusible CheY-P molecules function as the sole link between the input (chemoreceptors) and output (flagellum). The phosphatase (CheZ) limits the lifetime of CheY-P. The notable difference is the absence of the enzymes (CheR and CheB) involved in receptor-mediated adaptation, in *H. pylori*. Figure adapted from images created with BioRender.com.

The similarities in the mechanisms of flagellar modulation in *H. pylori* and *E. coli* are not surprising considering the existence of several shared components in the core chemotaxis network of the two species (53) (Figure 3). Similar to *E. coli*, *H. pylori* modulate flagellar reversals by phosphorylating CheY with the aid of CheA. *H. pylori* carry several types of membranous receptors (TlpA, B, C), in addition to a cytoplasmic receptor called TlpD. The phosphatase, CheZ, functions as a sink for CheY-P to control CheY-P lifetimes. Additional elements in *H. pylori* include ChePep, which interacts with CheZ near the cell poles, and FliY, which localizes at the motor (54, 55). The notable difference between these two systems is the absence of CheR and CheB homologs in *H. pylori*. Instead, *H. pylori* harbors three CheV homologs, which are implicated in chemotactic adaptation in *Bacillus subtilis* (56). More work is needed to determine whether CheV proteins are responsible for chemotactic adaptation in *H. pylori*.

Because *H. pylori* modulate their CW bias, one may wonder if they cannot undergo chemotaxis due to the predicted errors in the puller mode. Experimental observations suggest that this is not the case. In fact, compelling evidence demonstrates that *H. pylori* respond to a diverse range of chemo-effectors (57). *H. pylori* have been observed to rapidly accumulate around micropipettes or agar plugs that release chemo-effectors (57–63). Additionally, in vivo studies have implicated chemotactic migration in the occurrence of these pathogens in sites of gastric injury (64, 65). Evidently, *H. pylori* successfully counteract chemotaxis errors in the puller mode through a mechanism that remains unidentified at present.

## FUTURE DIRECTIONS

*H. pylori* are microaerophilic, Gram-negative bacteria that have successfully colonized most of the world population. They are difficult to eradicate from an infected human and are implicated in peptic ulcers and non-cardia gastric cancers (66–68). Chemotaxis and motility appear crucial in helping these bacteria target favorable chemical habitats in the host (53). However, several aspects of how the signaling network modulates flagellar functions to achieve chemotaxis remain unknown.

Evidently, the *H. pylori* network modulates the probability of CW rotation, at least in response to the attractant urea (6). How do they solve the chemotaxis errors predicted by the canonical framework? Perhaps they employ unknown feedback mechanisms to truncate the puller mode during an inappropriate backward run. A combination of modeling and experimental studies could help uncover the type of feedback necessary to achieve chemotaxis in this species. In particular, computational studies at a single-cell level could help test the various assumptions made during error predictions. Experimental studies could probe whether *H. pylori* dispense with adaptation through dynamic modifications of the steepness of the CW bias versus [CheY-P] relationship, or whether they modulate additional flagellar functions, including rotational speeds. Considering the absence of CheR and CheB homologs, numerous questions remain regarding the functional roles of CheV proteins in *H. pylori*. Experimentally addressing these questions, and determining whether *H. pylori* undergo different types of flagellar transitions, for example, the wrapped flagellar mode, will be crucial. In this regard, developing innovative microfluidic devices and single-motor assays will be a significant leap forward in shaping future research.

## SUMMARY POINTS

1. Chemotaxis is well-studied in *Escherichia coli* but poorly understood in other bacterial species, particularly those that localize their flagella at the cell poles.
2. The canonical chemotaxis framework predicts errors in bacteria capable of swimming backward or using the puller mode, especially when there is no response adaptation.
3. These errors would be resolved if the chemotaxis network were to modulate reversal frequencies, instead of the probability of being in the puller mode.
4. Although some bacterial species modulate reversal frequencies, current evidence in *Helicobacter pylori* suggests that their network modulates puller mode probability.
5. To examine whether *H. pylori* employ a novel feedback mechanism in chemotaxis, computational and experimental studies of single-swimmer responses to chemical fields are needed.

## Abbreviations

CCW: counterclockwise rotation of the flagella as seen by an observer behind the cell
Run/pusher: mode of motility when rotation of flagella propels the cell in a relatively straight path, with flagella lagging behind
CW: clockwise direction of flagellar rotation as seen by an observer behind the cell
CW bias: fraction of time that a flagellar motor rotates CW
Chemo-effector: a ligand sensed by one of the chemoreceptors in the cell
Attractant: a chemo-effector that is preferred by the cell
Repellent: a chemo-effector that is not preferred by the cell
Backward run/puller: mode of motility when rotation of flagella propels the cell in a relatively straight path, with flagella leading the cell body
τ_pusher_: mean run time in the pusher mode
τ_pusher_: mean run time in the puller mode
Reversal frequency: number of times the cell alternates between pusher and puller modes in unit time

## References

[1] Miyata M, Robinson RC, Uyeda TQP, Fukumori Y, Fukushima S, 2020. Tree of motility—a proposed history of motility systems in the tree of life. Genes Cells 25(1):6–21 31957229 10.1111/gtc.12737 PMC7004002

[2] Henrichsen J 1972. Bacterial surface translocation: a survey and a classification. Bacteriol. Rev 36(4):478–503 4631369 10.1128/br.36.4.478-503.1972 PMC408329

[3] Jarrell KF, McBride MJ. 2008. The surprisingly diverse ways that prokaryotes move. Nat. Rev. Microbiol 6(6):466–76 18461074 10.1038/nrmicro1900

[4] Shigematsu M, Meno Y, Misumi H, Amako K. 1995. The measurement of swimming velocity of *Vibrio cholerae* and *Pseudomonas aeruginosa* using the video tracking methods. Microbiol. Immunol 39(10):741–44 8577263 10.1111/j.1348-0421.1995.tb03260.x

[5] Berg HC, ed. 2004. E. coli in Motion. New York: Springer

[6] Antani JD, Sumali AX, Lele TP, Lele PP. 2021. Asymmetric random walks reveal that the chemotaxis network modulates flagellar rotational bias in *Helicobacter pylori*. eLife 10:e63936 33493107 10.7554/eLife.63936 PMC7834020

[7] Josenhans C, Suerbaum S. 2002. The role of motility as a virulence factor in bacteria. Int. J. Med. Microbiol 291(8):605–14 12008914 10.1078/1438-4221-00173

[8] Liu R, Ochman H. 2007. Origins of flagellar gene operons and secondary flagellar systems. J. Bacteriol 189(19):7098–104 17644605 10.1128/JB.00643-07 PMC2045201

[9] Haiko J, Westerlund-Wikström B. 2013. The role of the bacterial flagellum in adhesion and virulence. Biology 2(4):1242–67 24833223 10.3390/biology2041242 PMC4009794

[10] Nakamura S, Minamino T. 2019. Flagella-driven motility of bacteria. Biomolecules 9(7):279 31337100 10.3390/biom9070279 PMC6680979

[11] Faguy DM, Jarrell KF. 1999. A twisted tale: the origin and evolution of motility and chemotaxis in prokaryotes. Microbiology 145(2):279–81 10075408 10.1099/13500872-145-2-279

[12] Berg HC. 1993. Random Walks in Biology. Princeton, NJ: Princeton Univ. Press

[13] Bourret RB, Stock AM. 2002. Molecular information processing: lessons from bacterial chemotaxis. J. Biol. Chem 277(12):9625–28 11779877 10.1074/jbc.R100066200

[14] Parkinson JS. 1993. Signal transduction schemes of bacteria. Cell 73(5):857–71 8098993 10.1016/0092-8674(93)90267-t

[15] Lowe G, Meister M, Berg HC. 1987. Rapid rotation of flagellar bundles in swimming bacteria. Nature 325(6105):637–40

[16] Darnton NC, Turner L, Rojevsky S, Berg HC. 2007. On torque and tumbling in swimming *Escherichia coli*. J. Bacteriol 189(5):1756–64 17189361 10.1128/JB.01501-06 PMC1855780

[17] Berg HC, Brown DA. 1972. Chemotaxis in *Escherichia coli* analysed by three-dimensional tracking. Nature 239(5374):500–4 4563019 10.1038/239500a0

[18] Falke JJ, Hazelbauer GL. 2001. Transmembrane signaling in bacterial chemoreceptors. Trends Biochem. Sci 26(4):257–65 11295559 10.1016/s0968-0004(00)01770-9 PMC2895674

[19] Khan S, Macnab RM. 1980. The steady-state counterclockwise/clockwise ratio of bacterial flagellar motors is regulated by protonmotive force. J. Mol. Biol 138(3):563–97 6774099 10.1016/s0022-2836(80)80018-0

[20] Turner L, Samuel AD, Stern AS, Berg HC. 1999. Temperature dependence of switching of the bacterial flagellar motor by the protein Che^Y13DK106YW^. Biophys. J 77(1):597–603 10388784 10.1016/S0006-3495(99)76916-X PMC1300356

[21] Cluzel P, Surette M, Leibler S. 2000. An ultrasensitive bacterial motor revealed by monitoring signaling proteins in single cells. Science 287(5458):1652–55 10698740 10.1126/science.287.5458.1652

[22] Lele PP, Shrivastava A, Roland T, Berg HC. 2015. Response thresholds in bacterial chemotaxis. Sci. Adv 1(9):e1500299 26601280 10.1126/sciadv.1500299 PMC4646794

[23] Yuan J, Branch RW, Hosu BG, Berg HC. 2012. Adaptation at the output of the chemotaxis signalling pathway. Nature 484(7393):233–36 22498629 10.1038/nature10964 PMC3335734

[24] Block SM, Segall JE, Berg HC. 1982. Impulse responses in bacterial chemotaxis. Cell 31(1):215–26 6760985 10.1016/0092-8674(82)90421-4

[25] Wadhams GH, Armitage JP. 2004. Making sense of it all: bacterial chemotaxis. Nat. Rev. Mol. Cell Biol 5(12):1024–37 15573139 10.1038/nrm1524

[26] Sourjik V, Wingreen NS. 2012. Responding to chemical gradients: bacterial chemotaxis. Curr. Opin. Cell Biol 24(2):262–68 22169400 10.1016/j.ceb.2011.11.008 PMC3320702

[27] Lele PP, Branch RW, Nathan VSJ, Berg HC. 2012. Mechanism for adaptive remodeling of the bacterial flagellar switch. PNAS 109(49):20018–22 23169659 10.1073/pnas.1212327109 PMC3523824

[28] Zhang C, He R, Zhang R, Yuan J. 2018. Motor adaptive remodeling speeds up bacterial chemotactic adaptation. Biophys. J 114(5):1225–31 29539407 10.1016/j.bpj.2018.01.018 PMC5883565

[29] Ishihara A, Segall JE, Block SM, Berg HC. 1983. Coordination of flagella on filamentous cells of *Escherichia coli*. J. Bacteriol 155(1):228–37 6345503 10.1128/jb.155.1.228-237.1983 PMC217673

[30] Taylor BL, Koshland DE. 1974. Reversal of flagellar rotation in monotrichous and peritrichous bacteria: generation of changes in direction. J. Bacteriol 119(2):640–42 4605064 10.1128/jb.119.2.640-642.1974 PMC245654

[31] Theves M, Taktikos J, Zaburdaev V, Stark H, Beta C. 2013. A bacterial swimmer with two alternating speeds of propagation. Biophys. J 105(8):1915–24 24138867 10.1016/j.bpj.2013.08.047 PMC3797586

[32] Thormann KM, Beta C, Kühn MJ. 2022. Wrapped up: the motility of polarly flagellated bacteria. Annu. Rev. Microbiol 76:349–67 35650667 10.1146/annurev-micro-041122-101032

[33] Duffy KJ, Ford RM. 1997. Turn angle and run time distributions characterize swimming behavior for *Pseudomonas putida*. J. Bacteriol 179(4):1428–30 9023235 10.1128/jb.179.4.1428-1430.1997 PMC178849

[34] Liu B, Gulino M, Morse M, Tang JX, Powers TR, Breuer KS. 2014. Helical motion of the cell body enhances *Caulobacter crescentus* motility. PNAS 111(31):11252–56 25053810 10.1073/pnas.1407636111 PMC4128131

[35] Fujii M, Shibata S, Aizawa S-I. 2008. Polar, peritrichous, and lateral flagella belong to three distinguishable flagellar families. J. Mol. Biol 379(2):273–83 18455187 10.1016/j.jmb.2008.04.012

[36] Taguchi F, Shibata S, Suzuki T, Ogawa Y, Aizawa S-I, 2008. Effects of glycosylation on swimming ability and flagellar polymorphic transformation in *Pseudomonas syringae* pv. tabaci 6605. J. Bacteriol 190(2):764–68 18024523 10.1128/JB.01282-07 PMC2223687

[37] Stocker R 2011. Reverse and flick: hybrid locomotion in bacteria. PNAS 108(7):2635–36 21289282 10.1073/pnas.1019199108 PMC3041106

[38] Son K, Guasto JS, Stocker R. 2013. Bacteria can exploit a flagellar buckling instability to change direction. Nat. Phys 9(8):494–98

[39] Segall JE, Block SM, Berg HC. 1986. Temporal comparisons in bacterial chemotaxis. PNAS 83(23):8987–91 3024160 10.1073/pnas.83.23.8987 PMC387059

[40] Yang J, Chawla R, Rhee KY, Gupta R, Manson MD, 2020. Biphasic chemotaxis of *Escherichia coli* to the microbiota metabolite indole. PNAS 117(11):6114–20 32123098 10.1073/pnas.1916974117 PMC7084101

[41] Lovely PS, Dahlquist FW. 1975. Statistical measures of bacterial motility and chemotaxis. J. Theor. Biol 50(2):477–96 1094203 10.1016/0022-5193(75)90094-6

[42] Dickinson RB, Tranquillo RT. 1993. A stochastic model for adhesion-mediated cell random motility and haptotaxis. J. Math. Biol 31(6):563–600 8376918 10.1007/BF00161199

[43] Tindall MJ, Maini PK, Porter SL, Armitage JP. 2008. Overview of mathematical approaches used to model bacterial chemotaxis II: bacterial populations. Bull. Math. Biol 70(6):1570–607 18642047 10.1007/s11538-008-9322-5

[44] Arumugam G, Tyagi J. 2021. Keller-Segel chemotaxis models: a review. Acta Appl. Math 171:6

[45] Ford RM, Lauffenburger DA. 1991. Measurement of bacterial random motility and chemotaxis coefficients: II. Application of single-cell-based mathematical model. Biotechnol. Bioeng 37(7):661–7 18600657 10.1002/bit.260370708

[46] Ford RM, Harvey RW. 2007. Role of chemotaxis in the transport of bacteria through saturated porous media. Adv. Water Resour 30(6–7):1608–17

[47] Mattingly HH, Emonet T. 2022. Collective behavior and nongenetic inheritance allow bacterial populations to adapt to changing environments. PNAS 119(26):e2117377119 35727978 10.1073/pnas.2117377119 PMC9245662

[48] Taktikos J, Stark H, Zaburdaev V. 2013. How the motility pattern of bacteria affects their dispersal and chemotaxis. PLOS ONE 8(12):e81936 24391710 10.1371/journal.pone.0081936 PMC3876982

[49] Gro mann R, Peruani F, Bär M. 2016. Diffusion properties of active particles with directional reversal. New J. Phys 18(4):043009

[50] Xie L, Lu C, Wu X-L. 2015. Marine bacterial chemoresponse to a stepwise chemoattractant stimulus. Biophys. J 108(3):766–74 25650943 10.1016/j.bpj.2014.11.3479 PMC4317561

[51] Alirezaeizanjani Z, Gro mann R, Pfeifer V, Hintsche M, Beta C. 2020. Chemotaxis strategies of bacteria with multiple run modes. Sci. Adv 6(22):eaaz6153 32766440 10.1126/sciadv.aaz6153 PMC7385427

[52] Cai Q, Li Z, Ouyang Q, Luo C, Gordon VD. 2016. Singly flagellated *Pseudomonas aeruginosa* chemotaxes efficiently by unbiased motor regulation. mBio 7(2):e00013–16 27048795 10.1128/mBio.00013-16 PMC4817248

[53] Lertsethtakarn P, Ottemann KM, Hendrixson DR. 2011. Motility and chemotaxis in *Campylobacter* and *Helicobacter*. Annu. Rev. Microbiol 65:389–410 21939377 10.1146/annurev-micro-090110-102908 PMC6238628

[54] Howitt MR, Lee JY, Lertsethtakarn P, Vogelmann R, Joubert L-M, 2011. ChePep controls *Helicobacter pylori* infection of the gastric glands and chemotaxis in the *Epsilonproteobacteria*. mBio 2(4):e00098–11 21791582 10.1128/mBio.00098-11 PMC3143842

[55] Lertsethtakarn P, Howitt MR, Castellon J, Amieva MR, Ottemann KM. 2015. *Helicobacter pylori* CheZHP and ChePep form a novel chemotaxis-regulatory complex distinct from the core chemotaxis signaling proteins and the flagellar motor. Mol. Microbiol 97(6):1063–78 26061894 10.1111/mmi.13086 PMC4677328

[56] Rao CV, Glekas GD, Ordal GW. 2008. The three adaptation systems of *Bacillus subtilis* chemotaxis. Trends Microbiol. 16(10):480–87 18774298 10.1016/j.tim.2008.07.003 PMC3532902

[57] Huang JY, Sweeney EG, Guillemin K, Amieva MR. 2017. Multiple acid sensors control *Helicobacter pylori* colonization of the stomach. PLOS Pathog. 13(1):e1006118 28103315 10.1371/journal.ppat.1006118 PMC5245789

[58] Perkins A, Tudorica DA, Amieva MR, Remington SJ, Guillemin K. 2019. *Helicobacter pylori* senses bleach (HOCl) as a chemoattractant using a cytosolic chemoreceptor. PLOS Biol. 17(8):e3000395 31465435 10.1371/journal.pbio.3000395 PMC6715182

[59] Collins KD, Andermann TM, Draper J, Sanders L, Williams SM, 2016. The *Helicobacter pylori* CZB cytoplasmic chemoreceptor TlpD forms an autonomous polar chemotaxis signaling complex that mediates a tactic response to oxidative stress. J. Bacteriol 198(11):1563–75 27002127 10.1128/JB.00071-16 PMC4959281

[60] Croxen MA, Sisson G, Melano R, Hoffman PS. 2006. The *Helicobacter pylori* chemotaxis receptor TlpB (HP0103) is required for pH taxis and for colonization of the gastric mucosa. J. Bacteriol 188(7):2656–65 16547053 10.1128/JB.188.7.2656-2665.2006 PMC1428400

[61] Rader BA, Wreden C, Hicks KG, Sweeney EG, Ottemann KM, Guillemin K. 2011. *Helicobacter pylori* perceives the quorum-sensing molecule AI-2 as a chemorepellent via the chemoreceptor TlpB. Microbiology 157(9):2445–55 21602215 10.1099/mic.0.049353-0 PMC3352171

[62] Cerda O, Rivas A, Toledo H. 2003. *Helicobacter pylori* strain ATCC700392 encodes a methyl-accepting chemotaxis receptor protein (MCP) for arginine and sodium bicarbonate. FEMS Microbiol. Lett 224(2):175–81 12892880 10.1016/S0378-1097(03)00423-3

[63] Cerda OA, Núñez-Villena F, Soto SE, Ugalde JM, López-Solís R, Toledo H. 2011. *tlpA* gene expression is required for arginine and bicarbonate chemotaxis in *Helicobacter pylori*. Biol. Res 44(3):277–82 22688915

[64] Hanyu H, Engevik KA, Matthis AL, Ottemann KM, Montrose MH, Aihara E. 2019. *Helicobacter pylori* uses the TlpB receptor to sense sites of gastric injury. Infect. Immun 87(9):e00202–19 31262979 10.1128/IAI.00202-19 PMC6704605

[65] Aihara E, Closson C, Matthis AL, Schumacher MA, Engevik AC, 2014. Motility and chemotaxis mediate the preferential colonization of gastric injury sites by *Helicobacter pylori*. PLOS Pathog. 10(7):e1004275 25033386 10.1371/journal.ppat.1004275 PMC4102597

[66] Hansen S, Melby KK, Aase S, Jellum E, Vollset SE. 1999. *Helicobacter pylori* infection and risk of cardia cancer and non-cardia gastric cancer: a nested case-control study. Scand. J. Gastroenterol 34(4):353–60 10365894 10.1080/003655299750026353

[67] Uemura N, Okamoto S, Yamamoto S, Matsumura N, Yamaguchi S, 2001. *Helicobacter pylori* infection and the development of gastric cancer. N. Engl. J. Med 345(11):784–89 11556297 10.1056/NEJMoa001999

[68] Polk DB, Peek RM. 2010. *Helicobacter pylori*: gastric cancer and beyond. Nat. Rev. Cancer 10(6):403–14 20495574 10.1038/nrc2857 PMC2957472

